# Risk Factors for Delayed Extubation after Ventricular Septal Defect Closure: a Prospective Observational Study

**DOI:** 10.21470/1678-9741-2017-0031

**Published:** 2017

**Authors:** Divyakant Parmar, Ketav Lakhia, Pankaj Garg, Kartik Patel, Ritesh Shah, Jigar Surti, Jigar Panchal, Himani Pandya

**Affiliations:** 1 Department of Cardiac Anesthesia of the U. N. Mehta Institute of Cardiology and Research Center (affiliated to BJ Medical College, Ahmedabad), Gujarat, India; 2 Department of Cardiovascular and Thoracic Surgery of the U. N. Mehta Institute of Cardiology and Research Center (affiliated to BJ Medical College, Ahmedabad), Gujarat, India; 3 Department of Research of the U. N. Mehta Institute of Cardiology and Research Center (affiliated to BJ Medical College, Ahmedabad), Gujarat, India

**Keywords:** Heart Septal Defects, Ventricular, Cardiac Output, Low, Vasoactive Inotropic Score

## Abstract

**Objective:**

The objective of our study was to determine the feasibility of early extubation and to identify the risk factors for delayed extubation in pediatric patients operated for ventricular septal defect closure.

**Methods:**

A prospective, observational study was carried out at our Institute. This study involved consecutive 135 patients undergoing ventricular septal defect closure. Patients were extubated if feasible within six hours after surgery. Based on duration of extubation, patients were divided two groups: Group 1= extubation time ≤ 6 hours, Group 2= extubation time >6 hours.

**Results:**

A total of 99 patients were in Group 1 and 36 patients in Group 2. Duration of ventilation was 4.4±0.9 hours in Group 1 and 25.9±24.9 hours in Group 2 (*P*<0.001). Univariate analysis showed that young age, low weight, low partial pressure of oxygen, trisomy 21, multiple ventricular septal defect, high vasoactive inotropic score, transient heart block and low cardiac output syndrome were associated with delayed extubation. However, regression analysis revealed that only trisomy 21 (OR: 0.248; 95%CI: 0.176-0.701; *P*=0.001), low cardiac output syndrome (OR: 0.291; 95%CI: 0.267-0.979; *P*=0.001), multiple ventricular septal defect (OR: 0.243; 95%CI: 0.147-0.606; *P*=0.002) and vasoactive inotropic score (OR: 0.174 95%CI: 0.002-0.062; *P*=0.039) are strongest predictors for delayed extubation.

**Conclusion:**

Trisomy 21, low cardiac output syndrome, multiple ventricular septal defect and high vasoactive inotropic score are significant risk factors for delay in extubation. Age, weight, pulmonary artery hypertension, size of ventricular septal defect, aortic cross-clamp and cardiopulmonary bypass time did not affect early extubation.

**Table:** 

Abbreviations, acronyms & symbols				
ABG	= Arterial blood gases		MAP	= Mean arterial pressure
ACC	= Aortic cross-clamp		PaCO_2_	= Partial pressure of carbon dioxide
ACT	= Activated clotting time		PAH	= Pulmonary arterial hypertension
ASD	= Atrial septal defect		PDA	= Patent ductus arteriosus
CPAP	= Continuous positive airway pressure		PEEP	= Peek end expiratory pressure
CPB	= Cardiopulmonary bypass		PH	= Pulmonary hypertension
ETCO_2_	= End-tidal CO_2_		PO_2_	= Partial pressure of oxygen
FiO_2_	= Fraction of inspired oxygen		PTFE	= Polytetrafluroethylene
ICU	= Intensive care unit		RHC	= Right heart catheterization
IS	= Inotropic score		VIS	= Vasoactive inotropic score
LCOS	= Low cardiac output syndrome		VSD	= Ventricular septal defect

## INTRODUCTION

In the inceptual era of pediatric cardiac surgery, prolonged mechanical ventilation was a norm in the postoperative care, which significantly contributed to morbidity and mortality. Early extubation (within 6-8 hours after surgery)^[1]^ following pediatric cardiac surgery was initially conceptualized in the 1980s^[2]^. This gradually received wide acceptance due to increasing number of patients and limited resources.

In the modern era, where chance of error tends to zero in terms of results, early extubation has improved results tremendously in appropriate cases by reducing potential complications of prolonged mechanical ventilation. This strategy reduces intensive care unit (ICU) and hospital stay, henceforth reduces overall treatment cost and utilization of resources.

In the literature, there have been relatively few articles addressing this useful strategy ^[3-10]^. But, despite of its advantages, it cannot be applied in all cases, as various factors decide whether early extubation is feasible or not. Therefore, we have performed a prospective observational study to identify the predictive factors for early extubation after ventricular septal defect (VSD) closure.

## METHODS

All consecutive patients operated for VSD closure between March 2015 and February 2016 in the Department of Cardiothoracic Surgery, U.N. Mehta Institute of Cardiology & Research Center, were enrolled in this prospective observational study. The study was approved by our institutional ethics committee and informed and written consent was obtained from the parents of all the patients. Inclusion criteria were presence of isolated or multiple VSDs, with or without atrial septal defect (ASD), and/or patent ductus arteriosus (PDA). Patients with associated complex congenital cardiac disease other than ASD and PDA and patients on ventilator preoperatively were excluded. Size of VSD was categorized echocardiographically as small, moderate or large. A large defect has a diameter of 75% or greater of the aortic annulus and ≤ 1 m/s of velocity flow by Doppler. A small defect has diameter < 33% of aortic annulus and a flow velocity ≥ 4 m/s^[11]^.

### Premedication and Anesthetic Management

Intravenous midazolam was used as premedication at dose 0.1 mg/kg, 15-20 minutes before induction of anesthesia. Patients were induced with intravenous fentanyl (5-10 µg/kg) and vacuronium (0.2 mg/kg). Inhalational induction with sevoflurane 8% in 100% oxygen was done in patients with no intravenous line. Maintenance anesthesia consisted of low concentration sevoflurane, intravenous fentanyl (1 µg/kg) and vacuronium 0.1 mg/kg hourly and whenever required.

Invasive blood pressure monitoring was performed through right radial or right femoral artery catheterization. Central venous pressure monitoring was performed through internal jugular vein catheterization. Other monitoring included electrocardiogram, oxygen saturation, end-tidal CO_2_ (ETCO_2_), nasopharyngeal temperature and urine output. Arterial blood gases (ABG), with haemoglobin, electrolytes, blood sugar, and activated clotting time (ACT) were also monitored. Monitoring during cardiopulmonary bypass (CPB) time included mean arterial pressure (MAP), urine output and systemic venous oxygen saturation. ABG and ACT were done half hourly while the patient was on pump.

### Surgery and Cardiopulmonary Bypass

All the patients were operated through midline sternotomy, moderate hypothermic cardiopulmonary bypass (temperature 32ºC) and blood cardioplegic arrest. VSD was closed with polytetrafluroethylene (PTFE) patch using continuous sutures or primarily using pledgeted sutures. Milrinone (50 µg/kg loading dose followed by 0.5 µg/kg/min infusion) and epinephrine (0.05 µg/kg/min) were used as a choice of inotropes while weaning from CPB and continued in the postoperative period. Inotropes were titrated depending upon hemodynamics. Milrinone was continued in the postoperative period at least 24 hours postextubation and gradually tapered thereafter. Before extubation, effect of muscle relaxant was reversed with glycopyrrolate (10 µg/kg) and neostigmine (0.05 mg/kg) intravenously. Postoperatively, all patients were given fentanyl infusion (1-2 µg/kg/hour) and intravenous paracetamol 15 mg/kg/dose 8 hourly for pain relief. Fentanyl infusion was continued until the patient was extubated and/or drains were removed.

### Criteria for Early Extubation

Patients were considered for extubation since they regained consciousness with adequate airway protective reflexes without the use of accessory respiratory muscle and if they met the following criteria: stable hemodynamics, adequate urine output, no significant mediastinal bleeding, adequate oxygenation at fraction of inspired oxygen (FiO_2_) 50% or less (pO_2_/FiO_2_ > 200), pH 7.35 or greater, partial pressure of carbon dioxide (PaCO_2_) in arterial blood ≤ 45 mmHg or less at peek end expiratory pressure (PEEP) 5 cmH_2_O on continuous positive airway pressure (CPAP) with pressure support ≤ 8 cmH_2_O. Intensivist, anesthesiologist and surgeon jointly decide on early or delayed extubation.

To identify the risk factors for early extubation, patients were divided into two groups based on duration of mechanical ventilation: Group 1 (≤ 6 hours) and Group 2 (> 6 hours). Both groups were compared for age, sex, weight, nutritional status, genetic disorders (*e.g.* Down syndrome), severity of pulmonary arterial hypertension (PAH), vasoactive inotropic score (VIS)^[12]^, type and number of VSDs, aortic cross-clamp (ACC) and CPB time, postoperative complications^[13]^, ICU stay, hospital stay and mortality. Definitive diagnosis of pulmonary hypertension (PH) requires an elevated mean pulmonary arterial pressure (P_pa_) of ≥ 25 mmHg at rest, measured by right heart catheterization (RHC).

Low cardiac output syndrome (LCOS) was defined as clinical signs or symptoms (tachycardia, oliguria, poor perfusion, cold extremities, hypotension, lowered level of consciousness, cardiac arrest) with or without a widened arterial-mixed venous oxygen saturation difference or metabolic acidosis ^[14,15]^. A stroke is any confirmed neurological deficit of abrupt onset caused by a disturbance in blood flow to the brain, when the neurologic deficit does not resolve within 24 hours. Significant bleeding (12 ml/kg/h in 1^st^ hour or 10 ml/kg/h in 2^nd^ hour or 8 ml/kg/h in the 3^rd^ hour) affecting the hemodynamics or requiring re-exploration ^[16]^. Hematologic dysfunction was defined as platelet count < 80,000/mm^[14]^ or a decline of 50% in the platelet count from the highest value recorded over the last 48 hours^[17]^. Hepatic dysfunction was defined as alanine transaminase level 2 times upper limit of normal. Prolonged ventilatory requirement was defined as need for invasive ventilation for more than 48 hours^[17]^.

### Statistical Analysis

Statistical analysis was carried out using SPSS version 20.0 software (SPSS Inc., USA). Quantitative data was expressed as mean ± SD whereas qualitative data was expressed as percentage. The *P* value less than 0.05 was considered to be significant. Univariate analysis of the continuous data using Student's t-test, whereas chi-square test was used for the categorical data. The variables following non-normal distribution were assessed using Mann-Whitney U test.

## RESULTS

Study included total 135 patients operated for VSD closure with or without ASD and/or PDA. Group 1 included 99 (73.3%) patients while Group 2 included 36 (26.7%). Demographic details of the patients are shown in Table 1. Mean age was 26.7 months (range 2 months to 12 years) and mean weight was 7.46 kg (range 3.4 to 25 kg). Trisomy 21 (Down syndrome) was present in 9 (6.6%) patients. One hundred and twenty (88.8%) patients had chronic malnutrition. However, mean age (26.37 months in Group 1 and 18.5 months in Group 2, *P*=0.019) was significantly more in Group 1 while incidence of trisomy 21 was significantly less (*P*=0.015) in Group 1 compared to Group 2. There was no significant difference between the two groups with regards to sex, type and size of VSD, severity of PAH and associated cardiac lesions. Incidence of multiple VSD was significantly more in Group 2 (*P*=0.019).

**Table 1:** Demographic details.

	Group 1 ≤ 6 hrs (n=99)	Group 2 > 6 hrs (n=36)	*P*value
Age (months)	26.37 ± 30.89	18.5 ± 26.2	0.019
Sex (male)	(56.56%)	(50%)	0.7869
Weight (kg)	7.85 ± 4.14	6.38 ± 3.68	0.005
Malnutrition	88 (87.8%)	32 (91.6%)	0.808
Trisomy 21 (n, %)	2 (3%)	7 (16.6%)	0.015
Type of VSD			
Perimembranous (n, %)	68 (68.6%)	20 (55.5%)	0.225
Subaortic (n, %)	19 (19.1%)	10 (27.7%)	0.402
Muscular (n, %)	12 (12.1%)	6 (16.6%)	0.688
Size of VSD			
Small VSD (n, %)	24 (25.2%)	7 (16.6%)	0.802
Moderate VSD (n, %)	4 (4.04%)	__	0.533
Large VSD (n, %)	72 (70.7%)	28 (83.3%)	0.480
PAH			
Mild PAH (n, %)	20 (21.21%)	3 (5.5%)	0.198
Moderate PAH (n, %)	10 (10.10%)	3 (8.3%)	0.931
Severe PAH (n, %)	70 (68.68%)	29 (86.1%)	0.208
Lesions			
Single VSD (n, %)	80 (79.79%)	23 (66.6%)	0.139
Multiple VSD (n, %)	5 (5.05%)	7 (19.4%)	0.019
VSD+PDA (n, %)	3 (3.03%)	1 (2.7%)	0.618
VSD+ASD (n, %)	12 (12.12%)	4 (11.1%)	0.888

VSD=ventricular septal defect; ASD=atrial septal defect; PAH=pulmonary arterial hypertension; PDA=patent ductus arteriosus

In intraoperative data, ACC time (*P*=0.443) and CPB time (*P*=0.522) were comparable in both groups (Table 2). Postoperatively, VIS was significantly higher in Group 2 (*P*=0.001). Arterial blood gas data was comparable in both groups, except arterial partial pressure of oxygen (PO_2_), which was significantly higher in Group 1 (*P*=0.034). Mean duration of mechanical ventilation (4.4±0.9 hours *vs.* 25.9±24.9 hours, *P*<0.001), ICU stay (3.1±1.3 days *vs.* 5.6±2.9 days, *P*<0.001), and hospital stay (7.8±2.4 days *vs.* 11.2±4.9 days, *P*<0.001) were significantly lower in Group 1 compared to Group 2.

**Table 2:** Intraoperative and postoperative data.

	Group 1≤ 6 hrs (n=99)	Group 2> 6 hrs (n=36)	*P* value
ACC time (min)	28.45±11.18	31.30±14.91	0.443
CPB time (min)	46.75±13.84	50.91±19.53	0.522
VIS	9.01±2.05	10.65±2.94	0.001
Arterial blood gas			
pH	7.38±0.04	7.38±0.07	0.937
PCO_2_ (mmHg)	38.29±5.78	38.20±7.9	0.978
PO_2_ (mmHg)	246.57±79.28	207.98±82.52	0.034
Hematocrit (%)	39.26±5.94	37.08±6.86	0.057
Bicarbonate (mEq/L)	26.24±24.82	22.85±3.12	0.893
MVT (hours)	4.4±0.9	25.9±24.9	<0.001
ICU stay (days)	3.1±1.3	5.6±2.9	<0.001
Hospital stay (days)	7.8±2.4	11.2±4.9	<0.001

ACC=aortic cross-clamp; CPB=cardiopulmonary bypass; VIS=vasoactive inotropic score; PCO2=partial pressure of carbon dioxide; PO2=partial pressure of oxygen; MVT=mechanical ventilation time; ICU=intensive care unit

Reintubation was required in 6 patients, of which 2 patients were in Group 1. Incidence of reintubation and postoperative complications, including transient heart block, LCOS, sepsis, significant bleeding and neurological events was greater in Group 2. However, the difference was not statistically significant except for LCOS (Table 3). Incidence of LCOS was significantly higher in Group 2 (*P*<0.001). There was one death in Group 2 due to LCOS and no deaths in Group 1, however the difference was not statistically significant (*P*=0.596). Regression analysis is shown in Table 4. Regression analysis found independent association of trisomy 21 (OR: 0.248; 95% CI: 0.176-0.701; *P*=0.001), multiple VSD (OR: 0.243; 95% CI: 0.147-0.606; *P*=0.002), VIS score (OR: 0.174; 95% CI: 0.002-0.062; *P*=0.039) and LCOS (OR: 0.291; 95% CI: 0.267-0.979; *P*=0.001) with late extubation. LCOS and trisomy 21 were the strongest predictor of late extubation, closely followed by multiple VSD and the least was VIS (Table 4).

**Table 3:** Postoperative complications and mortality.

	Group 1≤ 6 hrs (n=99)	Group 2>6 hrs (n=36)	*P*value
Reintubation	2 (2%)	4 (11.1%)	0.072
LCOS	0	6 (16.6%)	<0.001
Heart block			
Transient	__	3	
Permanent	__	__	
Renal dysfunction	__	__	
Hepatic dysfunction	__	__	
Respiratory failure	__	__	
Haematological complication	__	__	
Prolonged mechanical ventilation	__	__	
Sepsis	6 (6%)	2 (5.5%)	0.762
Bleeding	__	1 (2.7%)	0.596
Neurological event	__	1 (2.7%)	0.596
Mortality	__	1 (2.7%)	0.596

LCOS=low cardiac output syndrome

**Table 4:** Regression analysis.

	Unstandardized coefficients		Standardized coefficients			95% Confidence Interval for B	
	B	Std. Error	Beta	t	Sig.	Lower bound	Upper bound
(Constant)	0.199	0.193		1.030	0.305	-0.184	0.582
Age (month)	0.003	0.003	0.200	1.164	0.247	-0.002	0.008
Weight	-0.028	0.019	-0.261	-1.526	0.130	-0.065	0.008
PO_2_	-0.001	0.000	-0.144	-1.915	0.058	-0.002	0.000
Trisomy 21	0.438	0.133	0.248	3.303	0.001	0.176	0.701
Multiple VSD	0.376	0.116	0.243	3.240	0.002	0.147	0.606
VIS	0.032	0.015	0.174	2.090	0.039	0.002	0.062
Transient HB	0.211	0.267	0.071	0.790	0.431	-0.318	0.740
LCOS	0.623	0.180	0.291	3.462	0.001	0.267	0.979

VSD=ventricular septal defect; PO_2_=partial pressure of oxygen; VIS=vasoactive inotropic score; HB=heart block; LCOS=low cardiac output syndrome

## DISCUSSION

Advances in surgical and anaesthetic techniques have facilitated early extubation following paediatric cardiac surgery. Early extubation has led to decreased cardiac and respiratory morbidity, lower rate of nosocomial pneumonia, reduction in overall length of hospital stay and decreased cost of patient care^[8,18-20]^. Reintubation rate following early extubation is also reported to be very low (< 2-3%)^[21]^.

In our study, 74.1% patients were successfully extubated within 6 hours of surgery and mean duration of mechanical ventilation was 4.4±0.9 hours, while only 2% patients require reintubation after early extubation. These results are consistent with various studies. In the studies by Mittnacht et al.^[20]^ and Akhtar et al.^[17]^, 76% patients operated for VSD closure were successfully extubated early. Our results along with results of their studies show that with appropriate perioperative management, about 75% patients can be extubated early after VSD closure without significant increase in risk of reintubation.

In our study, on univariate analysis, age, weight, trisomy 21, multiple VSDs, high VIS, low PO_2_ and low cardiac output after surgery were significant risk factors for delayed extubation. However, on multivariate analysis, trisomy 21, multiple VSD, VIS score and LCOS after surgery were found to be independently associated with delayed extubation.

Patients who are operated for multiple, VSDs have higher risk of residual defect and ventricular dysfunction postoperatively, leading to increased mortality and morbidity^[23]^. Therefore, these patients are at higher risk for prolonged ventilation as seen in our study. Mean ventilation duration in these patients was significantly higher (13.7±8.5 hours *vs.* 4.4±0.9 hours) compared to patients with single VSD, despite having zero percent incidence of postoperative residual defect or ventricular dysfunction. However, we were able to extubate early 5 (41.6%) of the 12 patients with multiple VSDs. We feel that, although presence of multiple VSDs increases the risk of prolonged ventilation, it should not be taken as a contraindication to early extubation. Carefully selected patients with multiple VSDs can still be extubated early.

In our study, 9 (6.7%) patients had trisomy 21. Seven out of 9 patients had delayed extubation, however ACC and CPB time were comparable in patients with or without trisomy 21 (29.3±12.4 *vs.* 29.2±12.3, *P*=0.98 and 48±15.7 *vs.* 47.9±15.6, *P*=0.98 respectively). Of these 9 patients, none developed LCOS postoperatively. Our results are in congruence with Mittnacht et al.^[20]^. Studies have found that patients with trisomy 21 are at high risk for atelectasis and pneumonia^[20,21]^. We could not find any reason for prolonged intubation in these patients in our study.

LCOS was another significant risk factor for delayed extubation in our study. Shi et al.^[7]^, in their retrospective review of 172 infants operated for cardiac surgery, found that LCOS is an independent risk factor for prolonged ventilation and increased mortality. In our study, patients with LCOS had significantly increased duration of ventilation (45.5±57.0 hrs) as compared to patients without LCOS (8.15±7.91 hrs). Studies have also found that positive pressure ventilation is helpful in patients with LCOS by decreasing work of breathing, improving systemic oxygen delivery and by improving cardiac performance. Thus, positive pressure ventilation act as an important hemodynamic support in postoperative systolic ventricular dysfunction^[20,23]^. Studies have also shown that greater inotropic score (IS) and VIS are signiﬁcantly associated with delayed extubation and extubation failure^[1,18,21,23,24]^ due to associated hemodynamic instability^[16]^. Our results are comparable to studies in literature.

Various studies have found age, ACC time and CPB time as independent risk factors for delayed extubation^[1,8,26]^; however, in our study, age, weight, sex, ACC time, CPB time, size of VSD, preoperative pulmonary hypertension and presence of malnutrition were not significant risk factor for delayed extubation. The possible mechanism given for these were mainly respiratory complications leading to delayed extubation. Literature has shown that CPB time > 150 minutes has been found to be a significant risk factor for delayed extubation^[1]^, but in our study maximum CPB time was only 98 minutes, so the chances of pump related respiratory complications were lower. In their study, age < 6 months was strongly associated with delayed extubation, which was not the case in our study^[1]^.

Many studies have also found young age as an independent risk factor for delayed extubation after paediatric cardiac surgery^[1,8,18,19]^. The reasons cited for this are poor fatigue resistance and endurance of respiratory muscles and immature respiratory centre with poor response to hypoxia and hypercarbia^[18,19]^. Many other factors responsible for delayed extubation are low body weight, malnutrition, frequent infections and depressed immunity^[10,20,25,26]^. However, most of recent studies, including ours, have failed to find age related risk factors significant for delayed extubation^[2,27]^. Mittnacht et al.^[20]^ in their study extubated patients as young as 2 months. The authors found that other risk factors, in association with young age, increased the risk of delayed extubation rather than age alone. Moreover, authors commented that it is the resistance of anaesthetist and intensivist rather than age of the patient, which is responsible for delay in extubation in young patients.

Earlier, preoperative PAH was considered a major limiting factor for early extubation^[8,21,28]^ due to risk of pulmonary hypertensive crisis. However, recent studies have found early extubation to be helpful in these patients by reduced airway irritation and ventilator-associated complications, such as laryngotracheal trauma, pulmonary hypertensive crisis during endotracheal tube suctioning, mucous plugging of endotracheal tubes, barotrauma secondary to positive airway pressure ventilation, ventilator-associated pulmonary infections and atelectasis^[30-31]^. Herein, we also did not find preoperative PAH as a risk factor for delayed extubation, comparable to study by Vida et al.^[29]^.

We found that early extubation translates into significantly reduced ICU stay and hospital stay without increasing the risk of reintubation or mortality, similar to results of various studies ^[3-10]^. This not only helps in early mobilisation of the patient and reduces parental anxiety, but also reduces the cost of patient care and provides better utilisation of limited hospital resources.

We believe that the most important determinant for successful early extubation is the approach to all the patients as potential candidates for early extubation and then to optimize the perioperative events to achieve this goal. When treated in this manner, even neonates and young infants operated for VSD closure can be successfully extubated early.

## CONCLUSION

Every child with VSD closure is a potential candidate of early extubation. Early extubation after VSD closure is successful in the majority of infants and small children without increasing the risk of reintubation or mortality. The strongest independent risk factors delayed extubation are trisomy 21, LCOS, multiple VSDs and high VIS.

**Table:** 

Authors' roles & responsibilities	
DP	Substantial contributions to the conception or design of the work; acquisition, analysis, or interpretation of data for the work; final approval of the version to be published
KL	Revising the work critically for important intellectual content; final approval of the version to be published
PG	Substantial contributions to the conception or design of the work; acquisition, analysis, or interpretation of data for the work; drafting the work or revising it critically for important intellectual content; final approval of the version to be published
KP	Acquisition, analysis, or interpretation of data for the work; drafting the work or revising it critically for important intellectual content; final approval of the version to be published
RS	Substantial contributions to the conception or design of the work; final approval of the version to be publisheda
JS	Revising the work critically for important intellectual content; final approval of the version to be published
JP	Analysis, or interpretation of data for the work; final approval of the version to be published
HP	Interpretation of data for the work; final approval of the version to be published

## References

[1] Cheng DC (1998). Fast-track cardiac surgery: economic implications in
postoperative care. J Cardiothorac Vasc Anesth.

[2] Glenn JD, Don HF, Ebert PA, Cohen NH, Matthay MA (1980). Tracheal extubation after cardiac surgery in
children. Anesthesiology.

[3] Vricella LA, Dearani JA, Gundry SR, Razzouk AJ, Brauer SD, Bailey LL (2000). Ultra fast track in elective congenital cardiac
surgery. Ann Thorac Surg.

[4] Kloth RL, Baum VC (2002). Very early extubation in children after cardiac
surgery. Crit Care Med.

[5] Silva ZM, Perez A, Pinzon AD, Ricachinewsky CP, Rech DR, Lukrafka JL (2008). Factors associated with failure in ventilatory weaning of
children undergone pediatric cardiac surgery. Rev Bras Cir Cardiovasc.

[6] Alhashemi JA, Sharpe MD, Harris CL, Sherman V, Boyd D (2000). Effect of subarachnoid morphine administration on extubation time
after coronary artery bypass graft surgery. J Cardiothorac Vasc Anesth.

[7] Shi S, Zhao Z, Liu X, Shu Q, Tan L, Lin R (2008). Perioperative risk factors for prolonged mechanical ventilation
following cardiac surgery in neonates and young infants. Chest.

[8] Shekerdemian LS, Penny DJ, Novick W (2000). Early extubation after surgical repair of tetralogy of
Fallot. Cardiol Young.

[9] Davis S, Worley S, Mee RB, Harrison AM (2004). Factors associated with early extubation after cardiac surgery in
young children. Pediatr Crit Care Med.

[10] Harrison AM, Cox AC, Davis S, Piedmonte M, Drummond-Webb JJ, Mee RB (2002). Failed extubation after cardiac surgery in young children:
prevalence, pathogenesis, and risk factors. Pediatr Crit Care Med.

[11] Marwali EM, Budiwardhana N, Sastroasmoro S, Pudjiadi A, Haas NA (2013). Prediction model for length of intubation with assisted
mechanical ventilation in pediatric heart surgery. Intensive Care Med.

[12] Hornberger LK, Sahn DJ, Krabill KA, Sherman FS, Swensson RE, Pesonen E (1989). Elucidation of the natural history of ventricular septal defects
by serial Doppler color flow mapping studies. J Am Coll Cardiol.

[13] Davidson J, Tong S, Hancock H, Hauck A, Cruz E, Kaufman J (2012). Prospective validation of the vasoactive-inotropic score and
correlation to short-term outcomes in neonates and infants after
cardiothoracic surgery. Intensive Care Med.

[14] Wheeler DS1, Jeffries HE, Zimmerman JJ, Wong HR, Carcillo JA (2011). Sepsis in the pediatric cardiac intensive care
unit. World J Pediatr Congenit Heart Surg.

[15] Abuchaim DC, Bervanger S, Medeiros SA, Abuchaim JS, Burger M, Faraco DL (2010). Early extubation in the operating room in children after cardiac
heart surgery. Rev Bras Cir Cardiovasc.

[16] Sá MP, Nogueira JR, Ferraz PE, Figueiredo OJ, Cavalcante WC, Cavalcante TC (2012). Risk factors for low cardiac output syndrome after coronary
artery bypass grafting surgery. Rev Bras Cir Cardiovasc.

[17] Akhtar MI, Hamid M, Minai F, Wali AR, Anwar-Ul-Haq, Aman-Ullah (2014). Safety profile of fast-track extubation in pediatric congenital
heart disease surgery patients in a tertiary care hospital of a developing
country: an observational prospective study. J Anaesthesiol Clin Pharmacol.

[18] Sethi SK, Goyal D, Yadav DK, Shukla U, Kajala PL, Gupta VK (2011). Predictors of acute kidney injury post-cardiopulmonary bypass in
children. Clin Exp Nephrol.

[19] Polito A, Patorno E, Costello JM, Salvin JW, Emani SM, Rajagopal S (2011). Perioperative factors associated with prolonged mechanical
ventilation after complex congenital heart surgery. Pediatr Crit Care Med.

[20] Mittnacht AJ, Thanjan M, Srivastava S, Joashi U, Bodian C, Hossain S (2008). Extubation in the operating room after congenital heart surgery
in children. J Thorac Cardiovasc Surg.

[21] Székely A, Sápi E, Király L, Szatmári A, Dinya E (2006). Intraoperative and postoperative risk factors for prolonged
mechanical ventilation after pediatric cardiac surgery. Paediatr Anaesth.

[22] Hamilton BC, Honjo O, Alghamdi AA, Caldarone CA, Schwartz SM, Van Arsdell GS (2014). Efﬁcacy of evolving early-extubation strategy on early
postoperative functional recovery in pediatric open-heart surgery: a matched
case-control study. Semin Cardiothorac Vasc Anesth.

[23] Shekerdemian RL, Nicholas DG, Ackerman AD, Argent AC, Biagas K, Carcillo JA Jr., Dalton HJ (2008). Cardiorespiratory interactions in children with heart
disease. Rogers' textbook of pediatric intensive care.

[24] Shekerdemian L (2009). Perioperative manipulation of the circulation in children with
congenital heart disease. Heart.

[25] Li S, Zhang Y, Li S, Wang X, Zhang R, Lu Z (2015). Risk factors associated with prolonged mechanical ventilation
after corrective surgery for tetralogy of Fallot. Congenit Heart Dis.

[26] Traiber C, Piva JP, Fritsher CC, Garcia PC, Lago PM, Trotta EA (2009). Profile and consequences of children requiring prolonged
mechanical ventilation in three Brazilian pediatric intensive care
units. Pediatr Crit Care Med.

[27] Ip P, Chiu CS, Cheung YF (2002). Risk factors prolonging ventilation in young children after
cardiac surgery: impact of noninfectious pulmonary
complications. Pediatr Crit Care Med.

[28] López-Herce CJ, Leyton-Avilés P, Urbano-Villaescusa J, Cidoncha-Escobar E, Del Castillo-Peral PJ, Carrillo-Alvarez A (2008). Risk factors for prolonged mechanical ventilation after cardiac
surgery in children. Med Intensiva.

[29] Vida VL, Leon-Wyss J, Rojas M, Mack R, Barnoya J, Castañeda AR (2006). Pulmonary artery hypertension: is it really a contraindicating
factor for early extubation in children after cardiac
surgery?. Ann Thorac Surg.

[30] Nichols DG, Cameron DE, Greeley WJ, Lappe DG, Ungerleider RM, Wetzel RC (1995). Critical heart disease in infants and children.

[31] Stanger P, Lucas RV Jr, Edwards JE (1969). Anatomic factors causing respiratory distress in acyanotic
congenital cardiac diseases. Special reference to bronchial
obstruction. Pediatrics.

